# Lignin-Based/Polypyrrole Carbon Nanofiber Electrode With Enhanced Electrochemical Properties by Electrospun Method

**DOI:** 10.3389/fchem.2022.841956

**Published:** 2022-02-08

**Authors:** Zhou-Rui Hu, Dan-Dan Li, Tae-Hee Kim, Min-Seok Kim, Ting Xu, Ming-Guo Ma, Sun-Eun Choi, Chuanling Si

**Affiliations:** ^1^ Beijing Key Laboratory of Lignocellulosic Chemistry, Engineering Research Center of Forestry Biomass Materials and Bioenergy, Research Center of Biomass Clean Utilization, College of Materials Science and Technology, Beijing Forestry University, Beijing, China; ^2^ Department of Forest Biomaterials Engineering, College of Forest and Environmental Sciences, Kangwon National University, Chuncheon, South Korea; ^3^ Tianjin Key Laboratory of Pulp and Paper, Tianjin University of Science and Technology, Tianjin, China

**Keywords:** supercapacitors, lignin, electrostatic spinning, polypyrrole, film

## Abstract

Tailoring the structure and properties of lignin is an important step toward electrochemical applications. In this study, lignin/polypyrrole (PPy) composite electrode films with microporous and mesoporous structures were designed effectively by electrostatic spinning, carbonization, and *in situ* polymerization methods. The lignin can not only reduce the cost of carbon fiber but also increase the specific surface area of composite films due to the removal of carbonyl and phenolic functional groups of lignin during carbonization. Besides, the compact three-dimensional (3D) conductive network structures were constructed with PPy particles densely coated on the lignin nanofibers, which was helpful to improve the conductivity and fast electron transfer during the charging and discharging processes. The synthesized lignin carbon fibers/PPy anode materials had good electrochemical performance in 1 M H_2_SO_4_ electrolyte. The results showed that, at a current density of 1 A g^−1^, the lignin carbon nanofibers/PPy (LCNFs/PPy) had a larger specific capacitance of 213.7 F g^−1^ than carbon nanofibers (CNFs), lignin carbon nanofibers (LCNFs), and lignin/PPy fiber (LPAN/PPy). In addition, the specific surface area of LCNFs/PPy reached 872.60 m^2^ g^−1^ and the average pore size decreased to 2.50 nm after being coated by PPy. Therefore, the independent non-binder and self-supporting conductive film is expected to be a promising electrode material for supercapacitors with high performance.

## Introduction

Supercapacitors rely on electrode materials for charge storage, so electrode materials are the key part of the performance of supercapacitors (Choi et al., 2020; WulanSeptiani et al., 2020; Fu et al., 2021; Xu et al., 2021b; Liu et al., 2021a; Liu et al., 2021b; Du et al., 2022). Carbon materials, such as porous carbon (Li et al., 2019a), graphene (Zhou et al., 2020), carbon nanotubes (Fan et al., 2020), and ordered mesoporous carbon (Wang et al., 2018), are considered the most suitable electrode materials for supercapacitors due to their high specific surface area, developed pore structure, high electronic conductivity, and excellent stability (Chen et al., 2020a; Shang et al., 2020; Xu et al., 2020c). Unfortunately, strong van der Waals forces between graphene sheets tend to cause graphene sheets to accumulate and agglomerate (Xiong et al., 2020). And the biggest problem in the preparation of carbon nanotube composites is that carbon nanotubes are difficult to disperse effectively into the polymer matrix (Sahoo et al., 2010). Also, the process of ordered mesoporous carbon is complicated due to the use of various templates (Lin et al., 2015). However, porous carbon has gained wide raw material sources, low cost, well-developed pores, and an easy-to-control structure (Li et al., 2020). Moreover, the large amount of oxygen functional groups such as -OH and -COOH in these materials as another advantage provided interesting attention for better superior charge storage (Ding et al., 2021). Like the latest report, Xu et al. (2022) prepared dung beetle forewing carbon materials with a hierarchical porous structure, self-doped nitrogen, oxygen, and a large specific surface area, which obtained a specific capacitance of 348 F g^−1^. Wang et al. (2022) converted waste peach gum as a raw material into layered porous carbon doped with N, P, and O through impregnation and carbonization. The electrode exhibited excellent electrochemical performance (490 F g^−1^ under 1 A g^−1^) due to the synergistic effects of high specific surface area and multiple heteroatomic co-doping amounts.

Therefore, more and more attention has been paid to the preparation of porous carbon electrode materials for supercapacitors using biomass as precursors (Lian et al., 2018; Zhu et al., 2018; Yang et al., 2019; Liu et al., 2021g). Lignocellulosic biomass is one of the most abundant resources, which is a promising source of renewable energy (Du et al., 2019; Liu et al., 2020a; Liu et al., 2020b; Wang et al., 2020; Liu et al., 2021d; Xu et al., 2021a; Zhang et al., 2021a). Lignocellulosic biomass is mainly composed of cellulose,hemicelluloses, and lignin (Liu et al., 2021e; Liu et al., 2021f; Xu et al., 2020b; Liu et al., 2021h; Wang et al., 2021a). Among them, lignin as a kind of biomass carbon precursor is considered with broad application prospects due to its high carbon yield, large space for molecular structure modification, and abundant industrial sources (Dai et al., 2019; Li et al., 2019b; Chen et al., 2020b; Chen et al., 2020c; Dai et al., 2020; Xu et al., 2020a; Zhou et al., 2021; Park et al., 2022). In addition, researchers are committed to designing and manufacturing advanced lignin carbon fibers with high specific surface area, controllable porosity, and appropriate pore size using electrostatic spinning technology, and this technique has obvious advantages over other preparation methods in controlling the fiber inner diameter, surface morphology, and orientation degree (Qu et al., 2021; Thongsai et al., 2021). However, pure lignin has a low molecular weight and is not easily spinnable into fibers in practical applications (An et al., 2019). Therefore, high-molecular-weight polymers such as polyacrylonitrile (Szabó et al., 2021), poly(vinyl pyrrolidone) (Cao et al., 2020a), polyethylene oxide (Dallmeyer et al., 2010), and polyvinyl alcohol (Camiré et al., 2020) need to be added to the lignin solution to improve the viscosity and spinnability of the fiber preparation spinning solution. Furthermore, the mechanical strength of spun fibers can be improved by using high-molecular-weight polymers. Meanwhile, the introduction of conductive polymer into carbon fiber can generate more electrochemical active sites for a rapid charge–discharge conversion reaction, thus further improving the electrochemical performance (Zhang et al., 2017). Polypyrrole (PPy) is one of the most widely studied conductive polymers, which has a broad application prospect in supercapacitors due to its excellent energy storage capacity, easy synthesis, and high conductivity (Tian et al., 2019; Du et al., 2021; Yuan and Ma, 2021). Unfortunately, PPy as a supercapacitor electrode undergoes continuous expansion and contraction during the doping/de-doping process, which reduces its cyclic stability and electrochemical performance (Tian et al., 2021). Therefore, researchers used the strategy of depositing PPy on carbon-based materials to obtain enhanced capacitance performance in practical applications (Fan et al., 2014). For example, Li et al. (He et al., 2021) fabricated graphene/graphite/PPy composite fibers using a vertical alignment method, and the 3D microelectrode was helpful to improve electrochemical performance. Zhan et al. (2021) developed electrode materials with high capacitance (5,299 mF cm^−2^) and mechanical flexibility by synthesizing PPy *in situ* in cellulose nanofiber/sulfonated carbon nanotube composite hydrogel. In the literature, our group reviewed multifunctional lignin-based composite materials and nano-lignin materials for emerging applications (Deng et al., 2021; Ma et al., 2021a). Moreover, we prepared the flexible N-doped carbon nanotubes/MXene/PAN nanocomposite films with improved electrochemical properties via the electrostatic spinning method (Li et al., 2021).

In this paper, the method of preparing PPy-coated lignin carbon fiber composite films by electrostatic spinning, *in situ* chemical polymerization, and carbonization was proposed. Electrostatic spinning combining carbonization has the advantages of large specific surface area, uniform pore distribution, and low density, compared with the vacuum filtration method. In a three-electrode system, the high capacitance of the composite films electrode was 213.7 F g^−1^ at a current density of 1 A g^−1^. More importantly, biomass lignin provided a possibility as a low-cost self-supporting electrode material for energy storage devices.

## Experimental Section

### Materials

Lignin was purchased from Shandong Longli Biotechnology Co., Ltd. Pyrrole, polyacrylonitrile (PAN) (Mw = 150,000), and *N*,*N*-dimethylformamide (DMF) were purchased from Shanghai Macklin Biochemical Co., Ltd. Ammonium persulfate ((NH_4_)_2_S_2_O_8_) and urea (CH_4_N_2_O) were purchased from Beijing Chemical Plant Co., Ltd. All other chemicals were of analytical grade.

### Preparation of Electrospinning Solution

The spinning solution was obtained by stirring lignin and PAN (ratio: 0:1 and 1:4) in DMF solvent for 24 h at room temperature until completely dissolved. The precursory solution was transferred into a 5 ml syringe for electrospinning by using a voltage in the range of 15–17 kV and a distance of 13–15 cm from the needle tip to the aluminum foil collector. After spinning, the fibers were collected, and the two kinds of electrospun fiber membranes were named “PAN” and “LPAN,” respectively.

### Preparation of Lignin/PPy Filament Fiber

The PPy-coated nanofiber films were prepared using a simple *in situ* chemical polymerization. The above electrospun nanofiber LPAN film was dipped into a beaker containing an aqueous solution of 25 ml pyrrole (5 g L^−1^), which had been stirred for several minutes in advance. Then, after soaking for 3 h, 25 ml of (NH_4_)_2_S_2_O_8_ (0.2 moL L^−1^) solution was added dropwise, and holding at 0°C for 4 h. The sample was removed from the solution and rinsed with deionized water to remove PPy particles and residual reactants and dried in an oven for 4 h. The film containing PPy was named “LPAN/PPy.”

### Preparation of Lignin-Based Carbon Fiber/PPy Composites

**SCHEME 1:**
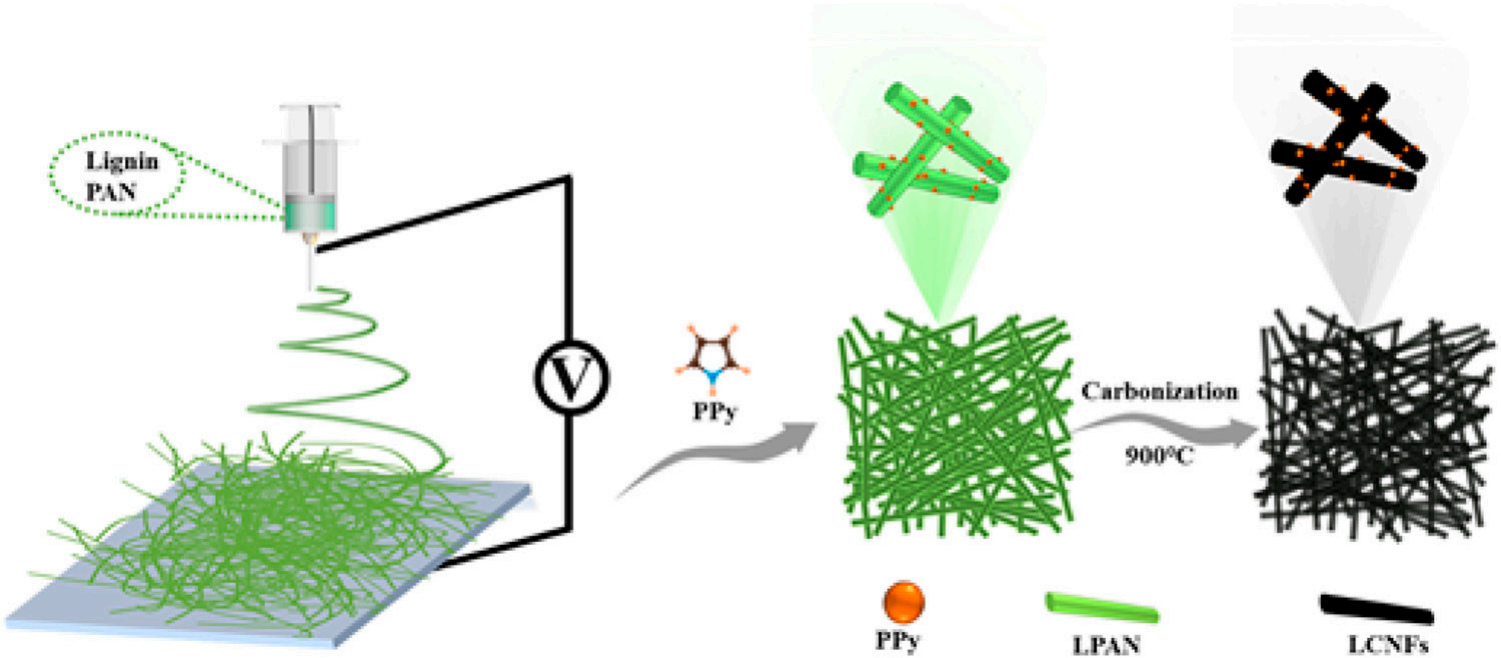
Schematic of the preparation of lignin-based carbon fiber/PPy composites.

The freestanding carbonized composites were fabricated as follows. Pyrolysis of polymer fibers was performed in a tubular furnace under the following condition: The heating rate was 1°C min^−1^ from room temperature to 250°C. The temperature was set constant at 250°C for 1 h and from 250 to 900°C with 5°C min^−1^ by blowing N_2_ gas. Then, the setup was maintained at that temperature for 2 h and cooled to room temperature. The preparation of lignin-based carbon fiber/PPy composites is shown in Scheme 1, which are named “LCNFs/PPy.” For comparison, the electrospun lignin-free carbon fiber film was prepared and marked as CNFs. In addition, the composites without adding PPy were prepared and marked as LCNFs.

### Material Characterization

The morphologies of the electrospun fiber membranes, lignin/PPy filament fibers, and lignin-based carbon fiber/PPy composites were characterized via scanning electron microscopy (SEM, SU8010, Hitachi, Japan). X-ray diffractometry (XRD, Ultima IV, Rigaku, Japan) was carried out to study the crystallographic information of the sample. TG-DTA (TG209F3, Netzsch, Germany) was tested under air to analyze the composition ration of the samples. The chemical groups were characterized with a PerkinElmer Frontier Fourier transform infrared (FT-IR) spectrometer.

### Electrochemical Measurements

All electrochemical tests were performed on an electrochemical workstation (CHI 660D) using a three-electrode configuration using a 1 M H_2_SO_4_ aqueous solution as the electrolyte at room temperature. A Pt mesh electrode and an Hg/HgCl_2_ electrode were used as the counter and reference electrodes, respectively. The cyclic voltammetry (CV) curves were plotted in a potential range between 0 and 1 V at different scan rates from 5 to 500 mV s^−1^. The EIS experiments in the frequency range of 1 MHz–0.01 Hz were executed at 5 mV AC amplitude. And the specific capacitance was calculated from galvanostatic charge/discharge (GCD) curves according to the following equation (Liu et al., 2021c):

$${\text{C}}_{s}=\frac{I\times \Delta t}{m\times \Delta U},$$ (1)

where *C*_*s*_ (F g^−1^) is the specific capacitance, *I* (A g^−1^) is the discharge current, *∆t* (s) is the discharge time, *∆U* (V) represents the potential window, and *m* (g) is the electrode material mass.

## Results and Discussion

**FIGURE 1:**
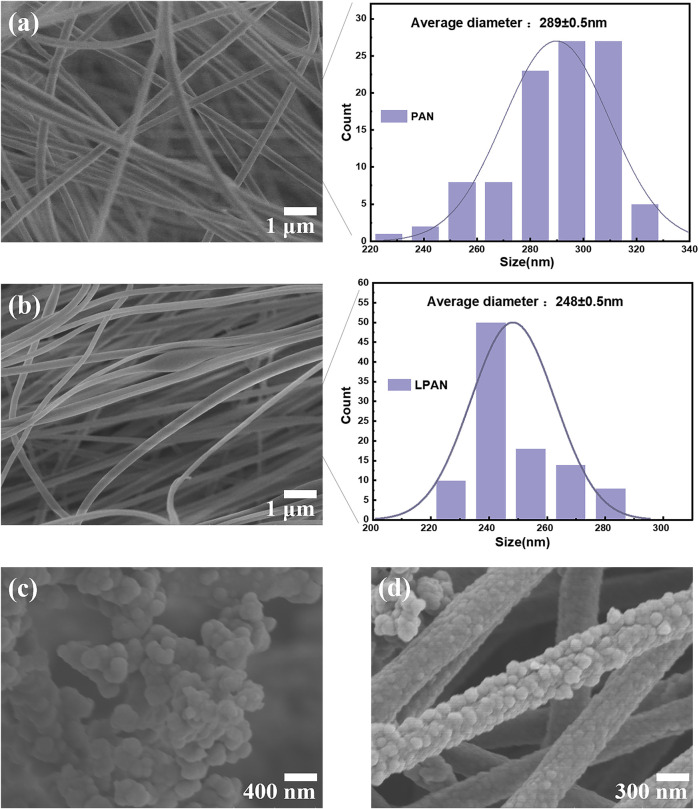
SEM images of electrospun fibers after thermal stabilization with fiber diameter distribution graphs: **(A)** stabilized PAN fibers; **(B)** stabilized LPAN fibers; **(C)** PPy; **(D)** LPAN/PPy.

Figure 1 shows the SEM images of the PAN, LPAN, PPy, and LPAN/PPy films prepared by electrostatic spinning and *in situ* chemical polymerization, which could intuitively reflect the microscopic morphology and structural differences of the precursor fibers. As shown in Figure 1A, the pure PAN fiber morphology was regular without beading or bending, which had uniform thickness and a diameter of about 289 nm. When the lignin:PAN ratio was 1:4, part of the fiber beaded and fractured (Figure 1B), and the fiber diameter was within the range of 248 nm. The interaction between lignin and PAN may contribute to the agglomerated, beading, and defective fibers. In addition, some lignin groups changed the polarity of the spinning solution, inducing the phenomenon of large fluctuation in the process of high-pressure spinning, which further affected the regularity of fiber diameter (Wang et al., 2013). Figure 1C shows the SEM image of PPy particles prepared by the chemical oxidation method. Besides, as shown in Figure 1D, the electrospinning fibers were coated with PPy evenly, and the diameter was mainly distributed at about 200–300 nm.

**FIGURE 2:**
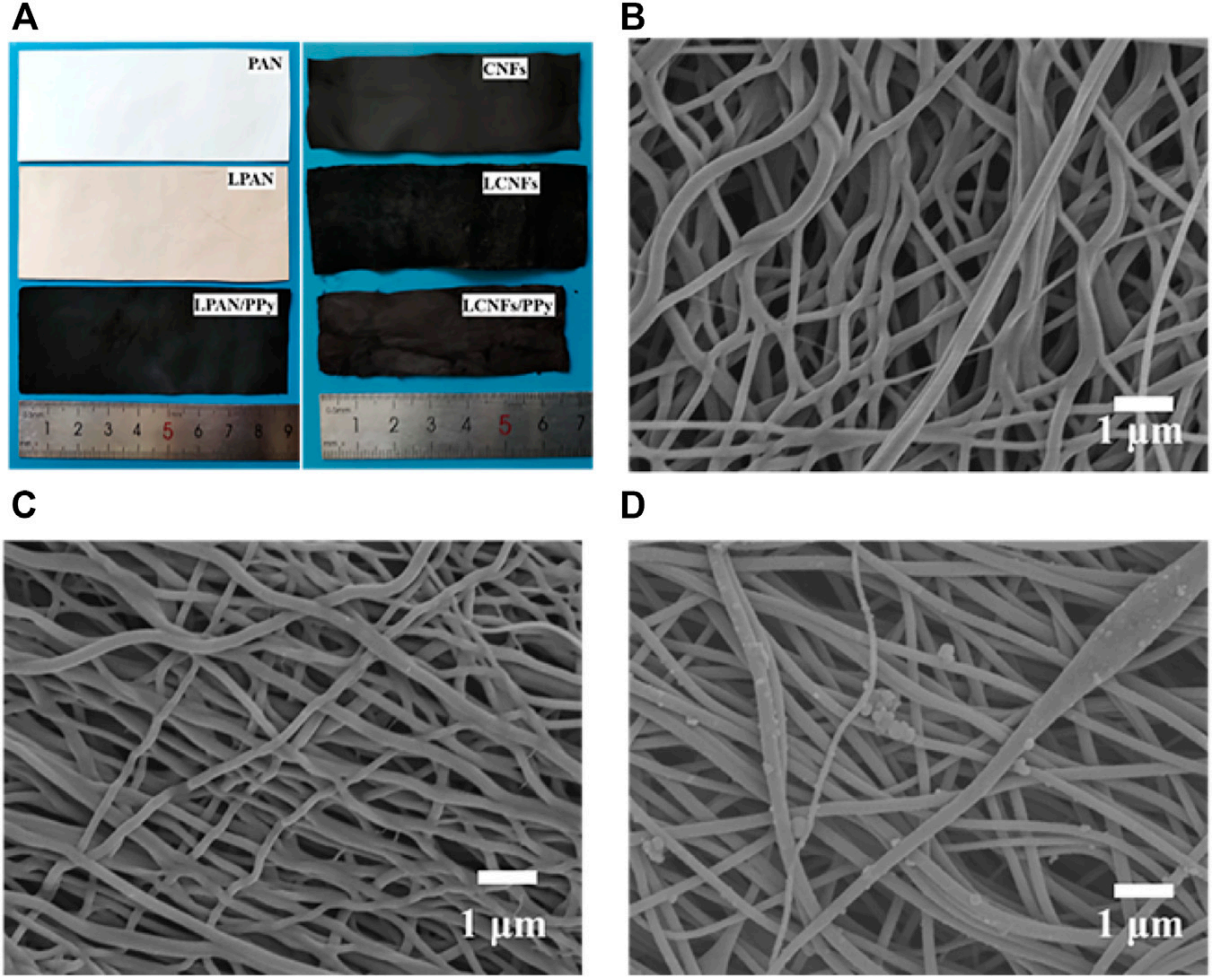
**(A)** Photographic image of the prepared samples. SEM micrographs of fibers after carbonization graphs: **(B)** CNFs; **(C)** LCNFs; **(D)** LCNFs/PPy.

Compared with lignin-based filament fibers prepared by the electrospinning method, the diameter of carbon fibers after pre-oxidation and high-temperature carbonization was significantly smaller, and part of the fiber showed a state of curvature and connection. It could be more intuitively observed from the digital image in Figure 2A that the sample area had a certain contraction after carbonization at 900°C. This could be attributed to the fracture, removal, and cyclization of lignin groups in the fiber (Ding et al., 2016; Ma et al., 2020). The LCNFs (Figure 2C) exhibited a smaller diameter than CNFs (Figure 2B), which increased the specific surface area and porosity, thereby improving the cyclic stability and specific capacitance. On the contrary, the carbon nanofibers connect and bridge with each other to form a highly conductive network structure that facilitated rapid electron transfer during charging and discharging processes, thus improving the rate capacity. In addition, PPy on the surface of carbon fiber was closely attached (Figure 2D), which also contributed to the improvement of electrochemical properties.

**FIGURE 3:**
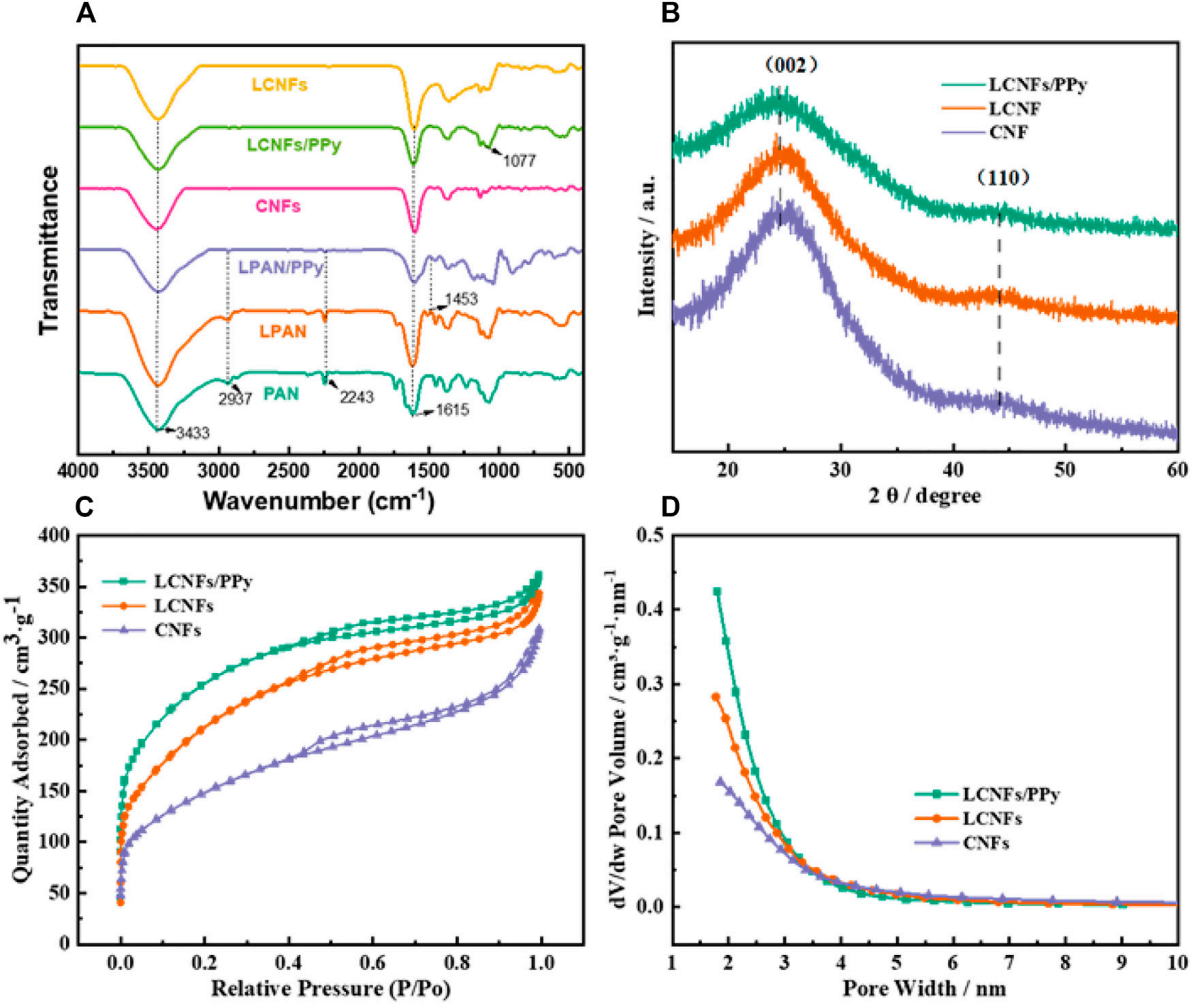
FT-IR spectra **(A)** of protofilament fibers and carbon fibers, XRD patterns **(B)**, N_2_ adsorption–desorption isotherms **(C)**, and pore-size distribution **(D)** of the carbon fiber composite electrodes.

FT-IR was performed to determine the chemical structure of the prepared samples (Figure 3A). The peak at 3,433 cm^−1^ was attributed to N-H in PAN, and O-H in the aromatic ring of lignin. In addition, the peaks of PAN at 2,937 and 2,243 cm^−1^ were attributed to C-H and C≡N, respectively (Si et al., 2009; Xu et al., 2021c). It was noted that the O-H peak increased with the increase of lignin content and had a trend of low-wavelength shift, and C≡N cyanine peaks gradually weakened. And the peaks at 1,183 and 1,077 cm^−1^ were C-N and C-H in a PPy long chain, respectively (Si et al., 2008; Wang et al., 2015). The results showed that the pyrrole rings were mainly connected by an α-α bond after the composite PPy on the surface of the filament. The FT-IR spectra of carbon fibers displayed three emblematic bands at 3,430 (N-H stretching), 1,610 (C=C stretching), and 1,370 cm^−1^ (-CH_3_ bending), respectively (Si et al, 2013; Ma et al., 2021b). After calcination at 900°C, the groups (cyanogenic C≡N in PAN) of the filament basically disappear, which was due to the decomposition of organic compounds at high temperature and the formation of amorphous carbon. The XRD patterns of carbon fibers are demonstrated in Figure 3B. The carbon peak position had been located at 26.6° and 44°, which corresponded to the (002) and (100) diffraction planes of disordered stacking of graphite structures (Jayachandran et al., 2021). It was also confirmed that the linear structure of the fiber was transformed into a heat-resistant trapezoidal structure during the pre-oxidation process, and the graphitization crystal structure could provide good structural stability, which was advantageous to improve the capacitive performance. In addition, a large specific surface area provided more active sites for charge storage, which improved the electrochemical performance of supercapacitors. The N_2_ adsorption–desorption isotherms and pore-size distribution of composite carbon fibers are illustrated in Figures 3C,D, respectively. According to IUPAC classification, the N_2_ adsorption–desorption isotherms of the three samples all exhibited a mixed type Ⅰ curve and type IV curve with a steep increase of N_2_ adsorbed at low pressure and a distinct hysteresis loop at high-pressure regions (0.4 < P/P_0_ < 1.0), indicating the coexistence of microporous and mesoporous structures (Schneidermann et al., 2017; Bai et al., 2020). The micropores increased the specific surface area of the material, increasing the active site of pseudo-capacitance reaction, and the mesopores provided a smooth channel to help electrolyte ions quickly enter the reaction interface of the material bulk phase. Therefore, the existence of these pores directly affected the specific capacity of the electrode material. Compared to CNFs (519.81 m^2^ g^−1^ and 3.55 nm), the BET surface area and pore diameter of LCNFs were 746.37 m^2^ g^−1^ and 2.76 nm, respectively. It was noted that the specific surface area of LCNFs/PPy reached 872.60 m^2^ g^−1^ and the average pore size decreased to 2.50 nm after the composite by PPy, which may be caused by the filling of the fiber gap with PPy to form smaller pores.

**FIGURE 4:**
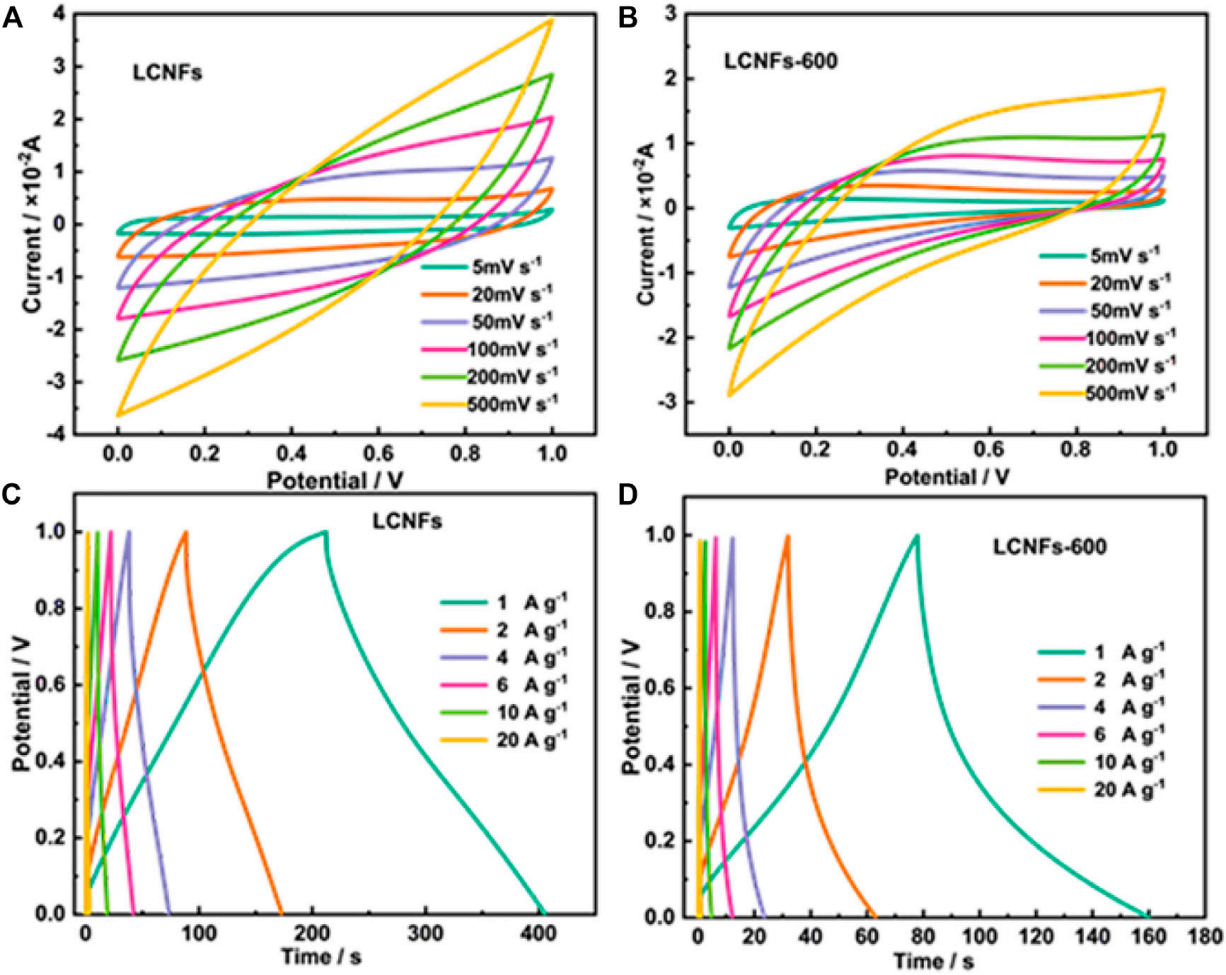
CV curves of LCNFs **(A)** and LCNFs-600 **(B)** at different scan rates with a potential range of 0–1 V. GCD cycle curves of LCNFs **(C)** and LCNFs-600 **(D)** with different charge densities.

To explore the electrochemical capacitive properties of carbon fibers at different temperatures, after that, the stabilized lignin/PAN fiber film was carbonized by heating to 600°C as the control sample, denoted as LCNFs-600. The capacitive properties of LCNF and LCNF-600 electrodes were measured in 1 M H_2_SO_4_ using a three-electrode system. From the electrode under different scan rate cyclic voltammetry (CV) curves, it is found that the curve area of the LCNFs (Figure 4A) was larger than that of the LCNFs-600 (Figure 4B). And at low scanning rates, the CV curves of LCNFs were closer to rectangles. These results clearly showed that the calcination temperature was 900°C and the graphitization and amorphous area of carbon fibers increased, further improving the specific surface area for better permeation H^+^ to access more active sites. Furthermore, at different current densities of 1–20 A g^−1^, the GCD curves of the LCNFs were near the isosceles triangle shapes (Figure 4C). Compared to LCNFs-600, the LCNF electrode had an ideal capacitance and ion adsorption/desorption mode during the energy storage process (Figure 4D).

**FIGURE 5:**
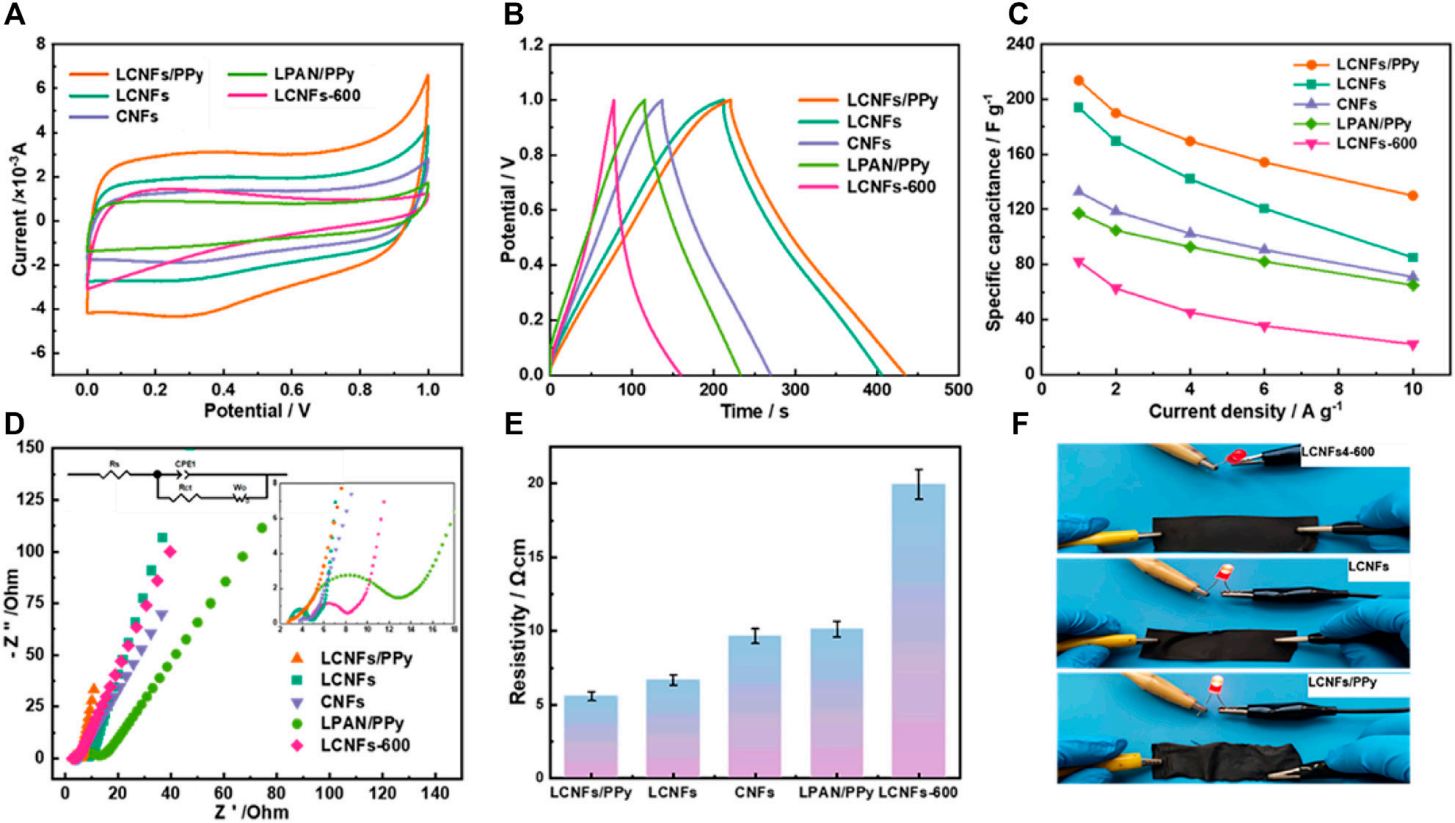
**(A)** CV curves at 5 mV s^−1^; **(B)** GCD curves at 1 A g^−1^; **(C)** specific capacitances at different current densities; **(D)** Nyquist plots (insets show a Randles cell equivalent circuit and zoomed plot of each curve); **(E)** conductivities of LCNFs/PPy, LPAN/PPy, LCNFs, LCNFs-600, and CNFs, respectively; **(F)** small bulb test.

Figure 5A shows the CV curves of composite electrode materials at 5 mV s^−1^. It was evident that the LCNF/PPy composite films approximated rectangles, which showed good electrochemical reversibility. Compared with LCNF composite films without PPy composite, the area was larger and the specific capacitance was higher, indicating that the addition of conductive polymer PPy could improve the specific capacitance of carbon fibers. In addition, with the addition of lignin, the charge storage capacity of the material significantly enhanced. This was attributed to the natural pore structure and complex functional groups of lignin, which enhanced the specific surface area and electrochemical reversibility of carbon fibers after calcination (Cao et al., 2020b). In order to better compare the electrochemical performance, chronopotentiograms are drawn for the samples at a constant current density of 1 A g^−1^ in Figure 5B. It could be observed that the LCNF/PPy composite film had a visibly larger discharging time (Δt) than the other samples. The relationships between specific capacitances and current densities of these five samples are shown in Figure 5C. The highest specific capacitance of 213.7 F g^−1^ was obtained for the LCNF/PPy electrode at a current density of 1 A g^−1^, compared with specific capacitances of 193.8 F g^−1^, 132.8 F g ^−1^ 117.3 F g^−1^, and 82.3 F g^−1^ for LCNFs, CNFs, LPAN/PPy, and LCNFs-600, respectively. And the LCNF/PPy electrode also showed a higher specific capacitance, compared with other recently reported lignin and PPy composite electrodes (Table 1). To further verify the excellent properties, EIS measurements were performed in the frequency range from 1 MHz to 0.01 Hz, as shown in Figure 5D. The series resistance of the LCNFs/PPy was only 2.7 Ω, and the small semicircle reveals the low charge transfer resistance (Rct). Moreover, it showed a high slope in the high-frequency region, which indicated good ion diffusion process and double-layer behavior in the electrochemical reaction process. Furthermore, the four-probe method was used to test the conductivity of the composite films, and the results are shown in Figure 5E. The addition of PPy into LCNFs has been demonstrated to be an effective strategy to reduce resistivity. The low resistivity of LCNFs/PPy (5.58 Ω cm^−1^) is also demonstrated in the small bulb test in Figure 5F. Moreover, the poor conductivity of the calcined sample at 600°C should be caused by its low graphitization degree and long charge diffusion path.

**TABLE 1:** Comparison of supercapacitive performance of recently reported lignin-based and PPy-based composite electrodes.

Electrode material	Specific capacitance	Electrolyte	Refs.
Lignin/PAN	148.0 F g^−1^ (50 mV s^−1^)	0.5 M KOH	Thongsai et al. (2021)
Lignin/LaMnO_3_	95.2 F g^−1^ (1 A g^−1^)	6 M KOH	Gang et al. (2021)
Lignin/KHCO_3_	114.0 F g^−1^ (0.5 A g^−1^)	2.5 M KNO_3_	Mutuma et al. (2021)
Lignin	197.3 F g^−1^ (0.2 A g^−1^)	6 M KOH	Sima et al. (2021)
Lignin	155.0 F g^−1^ (0.5 A g^−1^)	6 M KOH	Rong et al. (2021)
Alkali lignin	168.3 F g^−1^ (10 mV s^−1^)	3 M KCl	Rois et al. (2021)
PPy-thieno[3,4-b]thiophene	28.1 F g^−1^ (0.1 mA cm^−2^)	2 M LiCl	Wang et al. (2021b)
N,B-codoped graphene/PPy	160.3 F g^−1^ (0.5 A g^−1^)	1 M H_2_SO_4_	Xin et al. (2021)
PPy/birnessite	183.0 F g^−1^ (0.5 A g^−1^)	1 M Na_2_SO_4_	Zhuang et al. (2021)
Lignin/PPy	213.7 F g^−1^ (1 A g^−1^)	1 M H_2_SO_4_	This work

**FIGURE 6:**
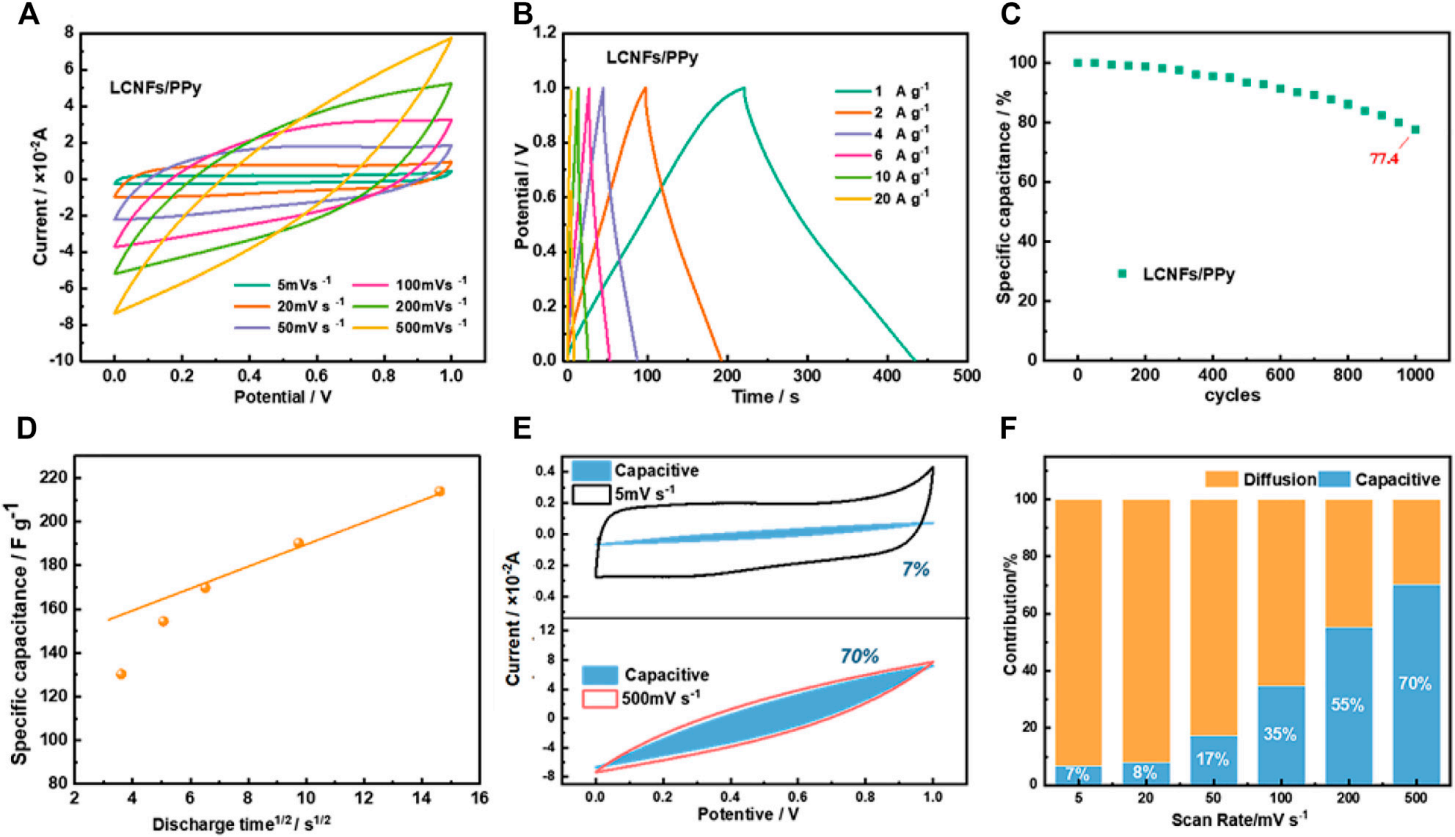
CV curves of LCNFs/PPy at different scanning rates **(A)**, GCD curves at different current densities **(B)**, cyclic performance of 1,000 charge–discharge cycles at current density 4 A g^−1^ **(C)**, relationship between the specific capacitance of LCNFs/PPy and discharge time **(D)**, capacitive contribution to charge storage at scan rates of 5 and 500 mV s^−1^ **(E)**, and percentage of capacitance contribution at different scan rates **(F)**.

To further clarify the electrochemical behavior of LCNF/PPy films, the complete CV curves and GCD curves of the SCs are plotted in Figures 6A,B, respectively. The results in Figure 6A showed that all CV curves maintain the similar shape at different scanning rates, demonstrating well capacitance performance and relatively good rate capability. Subsequently, the GCD curves of the LCNFs/PPy are displayed in Figure 6B. The curves had a shape of a symmetrical triangle that showed good capacitive behavior. It was important to assess the long-cycle stability of LCNF/PPy positive material by repeating the GCD test at 4 A g^−1^. Figure 6C shows a well stability of about 77% after 1,000 cycles. For further understanding the charge storage process of LCNFs/PPy, the electrochemical dynamics of electrode composite films were studied. The capacitance *C* could be calculated by (Lin et al., 2015)

$$\mathrm{C=}K_{1}+K_{2}T^{1/2},$$ (2)

where *T* is the discharge time of the GCD test, k_1_ corresponds to the surface capacitance effect (usually from the double-layer capacitance, *T*→0), and k_2_ *T* ^1/2^ corresponds to the capacitance effect of diffusion control (affected by the charge and discharge rates, *T*→∞). Figure 6D shows the relationship between specific capacitance and discharge time of LCNFs/PPy. When *T*→0, the intercept was k_1_, representing the specific capacitance contributed by the double-layer effect. The double-layer capacitance of LCNFs/PPy reached 148.7 F g^−1^, accounting for 69.6% of the total capacitance, which showed that the capacitance effect mainly came from double-layer adsorption (Chen et al., 2021a). In addition, the capacitance control and diffusion control in total charge storage could be further calculated and quantified by (Zhang et al., 2021b)

$$i=k_{1}v+k_{2}v^{\mathrm{1/2}}.$$ (3)

In short, at a certain voltage (v), the current response (*i*) consisted of two parts, wherein *k*_*1*_v and *k*_*2*_v^1/2^ corresponded to the surface control process (pseudo-capacitance and double-layer capacitance) and the diffusion control process, respectively (Chen et al., 2021b). As could be seen from Figures 6E,F, the capacitance contribution of the LCNF/PPy positive electrode film enlarged from 7 to 70% with the increase of scanning rate, which was caused by the relatively low ion diffusion rate at large scanning rates.

## Conclusion

In summary, carbon fiber precursors with lignin and PAN (ratio 1:4) were prepared by the electrostatic spinning method, and PPy was *in situ* polymerized to improve electrochemical performance. In addition, lignin/PPy composite films were produced without the use of any crosslinking agents and physical/chemical activation during thermal stabilization and carbonization. Lignin/PPy composite films with microporous and mesoporous structures were designed as the positive materials of the supercapacitor. Among them, the LCNF/PPy electrode had a large specific surface area, high pore volume, and the specific capacitance of 213.7 F g^−1^ at the current density of 1 A g^−1^. This work has the potential to use lignin to produce carbon fibers as a low-cost electrode material for high-performance supercapacitors.

## Data Availability

The original contributions presented in the study are included in the article/Supplementary Material, and further inquiries can be directed to the corresponding authors.
